# Risk of Sudden Sensorineural Hearing Loss in Patients with Common Preexisting Sensorineural Hearing Impairment: A Population-Based Study in Taiwan

**DOI:** 10.1371/journal.pone.0121190

**Published:** 2015-03-27

**Authors:** Malcolm Koo, Juen-Haur Hwang

**Affiliations:** 1 Department of Medical Research, Dalin Tzu Chi Hospital, Buddhist Tzu Chi Medical Foundation, Dalin, Chiayi, Taiwan; 2 Dalla Lana School of Public Health, University of Toronto, Ontario, Canada; 3 School of Medicine, Tzu Chi University, Hualien, Taiwan; 4 Department of Otolaryngology, Dalin Tzu Chi Hospital, Buddhist Tzu Chi Medical Foundation, Dalin, Chiayi, Taiwan; 5 Sleep Center, Dalin Tzu Chi Hospital, Buddhist Tzu Chi Medical Foundation, Dalin, Chiayi, Taiwan; Harvard University, UNITED STATES

## Abstract

**Objective:**

The role of preexisting sensorineural hearing impairment on the risk for sudden sensorineural hearing loss (SSHL) is still unclear. In this study, we aimed to assess the risk of SSHL in patients with common preexisting sensorineural hearing impairment using population-based data.

**Methods:**

A population-based case-control study design was used to analyze claims data between January 2001 and December 2011 obtained from the Taiwan National Health Insurance Research Database. The cases consisted of 514 patients with SSHL and the controls were frequency matched to 2,570 cases by sex, 10-year age group, and year of index date. Common sensorineural hearing impairments were retrospectively assessed in the cases and controls. Associations between sensorineural hearing impairment and risk of SSHL were evaluated using unconditional univariate and multivariate logistic regression analyses.

**Results:**

The mean age for the 3,084 study subjects was 53.1 years (standard deviation, S.D. = 15.6). Of the 514 cases, 49 (9.5%) had sensorineural hearing impairment while only 44 (1.7%) of the 2,570 controls had the same condition. Univariate logistic regression analyses indicated that preexisting sensorineural hearing impairment was significantly associated with SSHL (odds ratio, OR = 6.05, *p* < 0.001). Other comorbidities including hypertension, diabetes mellitus, and hyperlipidemia also showed significant associations with SSHL. Similar results were obtained when the association between SSHL and sensorineural hearing impairment was adjusted with either all the covariates (adjusted OR = 6.22, *p* < 0.001) or with only those selected using a backward elimination procedure (adjusted OR = 6.20, *p* < 0.001).

**Conclusions:**

Results from this population-based case-control study revealed that common sensorineural hearing impairment might be a novel risk factor for SSHL.

## Introduction

Sudden sensorineural hearing loss (SSHL) is defined as a loss of 30 dB or more in three contiguous frequencies over less than three days. It can be an isolated symptom or a presenting symptom of systemic diseases. The etiology and pathogenesis remain largely unknown [1,2]. A systematic review of 23 studies revealed that the suspected etiologies for SSHL was 71.0% for idiopathic cause, 12.8% for infectious diseases, 4.7% for otologic diseases, 4.2% for trauma, 2.8% for vascular or hematologic problems, 2.3% for neoplastic diseases, and 2.2% for other causes [3]. Other studies have reported increased risk of developing SSHL among patients with acquired and inherited cardiovascular risk factors [4], migraine [5], systemic lupus erythematosus [6], human immunodeficiency virus [7] and chronic kidney disease (CKD) [8]. Nevertheless, the role of preexisting sensorineural hearing impairment in SSHL has not been investigated except a retrospective study of 257 patients, in which no differences in remission rates for SSHL patients with preexisting sensorineural hearing loss was found [9]. Since a number of common etiologies can account for both of SSHL and common sensorineural hearing impairment, such as cardiovascular diseases [4, 10, 11], and CKD with diabetes mellitus (DM) [8], it is reasonable to hypothesize that common sensorineural hearing impairment may also increase the risk of SSHL. On the contrary, hypoxic preconditioning had been shown to protect some strains of mice from noise-induced hearing loss [12] and sound preconditioning could also inhibit ototoxic hearing loss in mice [13]. If this is indeed the case, it may be hypothesized that common sensorineural hearing impairment can decrease the risk of SSHL. Therefore, to gain a further understanding of the above possible and opposite hypotheses, we used data from a nationwide claims database to investigate the risk of SSHL in patients diagnosed with common sensorineural hearing impairment.

## Materials and Methods

This study was approved by the institutional review board of the Dalin Tzu Chi Hospital, Buddhist Tzu Chi Medical Foundation, Taiwan (No. B10202022). Since the NHIRD files contain only de-identified secondary data, the review board waived the requirement for obtaining informed consent from the patients.

### Study design and data source

The data for this nationwide population-based case-control study were obtained from the Longitudinal Health Insurance Database 2000 (LHID 2000), which is a subset database of the Taiwan National Health Insurance Research Database (NHIRD). The LHID 2000 contains medical services utilization information since 1996 for a randomly selected sample of one million beneficiaries registered in 2000, representing approximately 5% of Taiwan’s population. The Taiwan National Health Insurance is a universal single-payer compulsory health insurance program that instituted in March 1995 and it has a coverage of 99.9% of the Taiwan's population [14].

### Study sample and measurements

**Fig 1:**
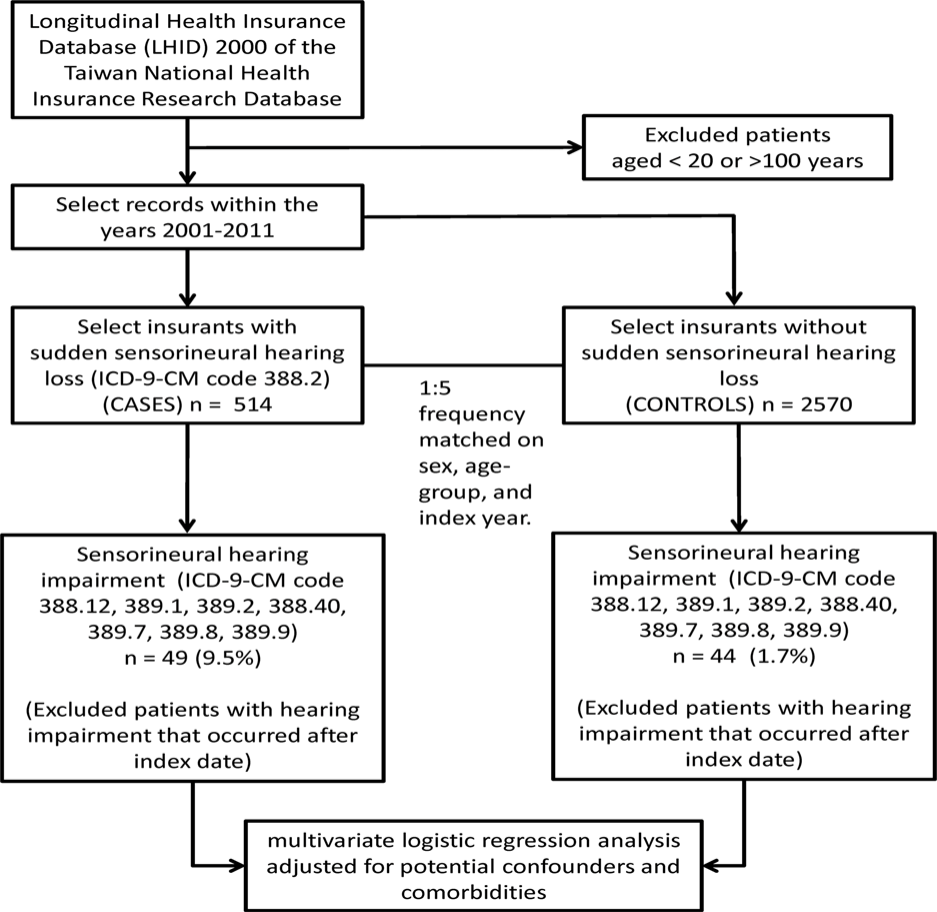
Flow diagram of the study. **ICD-9-CM.** International Classification of Diseases, Ninth Revision, Clinical Modification.

Both cases and controls, aged between 20 and 100 years, were identified from the LHID 2000 with records between January 1, 2001 and December 31, 2011. The case definition included three criteria: (1) patients who received at least two diagnoses of SSHL (International Classification of Diseases, 9^th^ Revision, Clinical Modification [ICD-9-CM] code 388.2), (2) the two diagnoses must occurred within 90 days or less, and (3) the diagnoses must be made by an otolaryngologist. Five controls per case were randomly selected from the LHID 2000, frequency matched by sex, age group (20–29, 30–39, 40–49, 50–59, 60–69, 70–79, and ≥ 80 years), and year of index date. Patients with conductive hearing loss (ICD-9-CM codes 389.0x), Ménière's disease (ICD-9-CM code 386.0), or vestibular schwannoma (ICD-9-CM code 225.1) were excluded from the study (Fig. 1).

The main outcome variable of common sensorineural hearing impairment in both cases and controls was assessed based on at least two diagnoses of ICD-9-CM code 388.12, 388.40, 389.1, 389.2, 389.7, 389.8, or 389.9 that occurred at least 30 days prior to the index date. In addition, these diagnoses must be made by an otolaryngologist. Other comorbidities included hypertension (ICD-9-CM codes 401.xx–405.xx), DM (ICD-9-CM code 250.xx), coronary artery disease (CAD) or myocardial infarction (MI) (ICD-9-CM codes 414.xx, 410.xx, and 429.xx), CKD (ICD-9-CM codes 585.xx and 586.xx), hyperlipidemia (ICD-9-CM codes 272.xx), and obesity (ICD-9-CM codes 278.0, 278.00, 278.01, and 278.02) were also assessed.

Urbanization of levels of the residence of cases and controls were constructed according to a published categorization scheme, which is based on a combination of population density, percentage of residents with college level or higher education, percentage of residents 65 years and older, percentage of residents who were agriculture workers, and the number of physicians per 100,000 people [15]. In addition, payroll-related insured amount was used as a proxy measure to represent socioeconomic status. The variable was categorized into tertiles with the lower and upper cut-points at New Taiwan $18,300 and 24,000, respectively.

### Statistical analysis

Distributions of age groups, sex, urbanization level of patient's residence, and tertile of insured amount between cases and controls were evaluated using Chi-square test. Unconditional univariate logistic regression analyses were used to evaluate the risks of SSHL associated with sensorineural hearing impairment and various comorbidities. In addition, two multivariate logistic regression models including different comorbidities were used to assess the independent association between SSHL and sensorineural hearing impairment. In Model 1, ten covariates including age, sex, urbanization level of patient's residence, tertile of insured amount, hypertension, DM, CAD or MI, CKD, hyperlipidemia, and obesity were included. Model 2 included only covariates that were obtained based on a backward elimination procedure using the *p*-value of the likelihood ratio test in the multivariate logistic regression analysis. All analyses were performed using IBM SPSS Statistics software package, version 22.0 (IBM Corp., Armonk, NY, USA). A *p* < 0.05 was considered statistically significant.

## Results

Table 1 presents the basic characteristics of the cases and controls. The mean age for the total 3,084 patients was 53.1 years (standard deviation, S.D. = 15.6). There were no significant differences in age and sex because of the use of frequency matching on these two variables. The distribution of the urbanization level of residence and the tertiles of insured amount between the cases were not significantly different compared with controls.

**Table 1:** Basic characteristics of patients with sudden sensorineural hearing loss and controls.

Variable	Frequency (%)		*p* value
	SSHL	Controls	
	(n = 514)	(n = 2,570)	
**Age** (years)			0.141
mean (std dev)	54.0 (15.8)	52.9 (15.6)	
minimum, maximum	20, 91	20, 96	
**Sex**			> 0.999
male	288 (56.0)	1,440 (56.0)	
female	226 (44.0)	1,130 (44.0)	
**Urbanization level of patient's residence** ^**1**^			0.204
1 (most urbanized)	146 (29.0)	730 (28.9)	
2	145 (28.8)	709 (28.1)	
3	78 (15.5)	496 (19.6)	
4	81 (16.1)	370 (14.6)	
5 (less urbanized)	53 (10.5)	221 (8.7)	
**Tertile of insured amount** ^**2**^			0.461
1 (lowest)	227 (44.2)	1,213 (47.2)	
2	157 (30.6)	736 (28.6)	
3 (highest)	129 (25.1)	620 (24.1)	

SSHL: sudden sensorineural hearing loss.

^1^Eleven cases and 44 controls had missing information on the urbanization levels and 1 case and 1 control had missing information on the insured amount.

^2^Lower and upper cut-points for the median insured amount are New Taiwan $18,300 and 24,000, respectively.

Table 2 shows the distribution of sensorineural hearing impairment and comorbidities between the cases and controls and also the results of the univariate logistic regression analyses. Of the 514 patients with SSHL, 49 (9.5%) had preexisting sensorineural hearing impairment while only 44 (1.7%) of the 2,570 controls had the same condition. Results from the univariate logistic regression analyses indicated that preexisting sensorineural hearing impairment was significantly associated with SSHL (OR = 6.05, *p* < 0.001). Other comorbidities including hypertension, DM, and hyperlipidemia also showed significant associations with SSHL.

**Table 2:** Univariate logistic regression of patients with sudden sensorineural hearing loss and controls.

Variable	Frequency (%)		Odds ratio (95% CI)	*p* value
	SSHL	Controls		
	(n = 514)	(n = 2,570)		
**Sensorineural hearing impairment**				
yes	49 (9.5)	44 (1.7)	6.05 (3.98–9.20)	< 0.001
no	465 (90.5)	2,526 (98.3)	1.00	
**Hypertension**				
yes	183 (35.6)	748 (29.1)	1.35 (1.10–1.64)	0.003
no	331 (64.4)	1,822 (70.9)	1.00	
**Diabetes mellitus**				
yes	101 (19.6)	366 (14.2)	1.47 (1.16–1.88)	0.002
no	413 (80.4)	2,204 (85.8)	1.00	
**CAD or myocardial infarction**				
yes	71 (13.8)	282 (11.0)	1.30 (0.98–1.72)	0.065
no	443 (86.2)	2,288 (89.0)	1.00	
**Chronic kidney disease**				
yes	19 (3.7)	60 (2.3)	1.61 (0.95–2.72)	0.077
no	495 (96.3)	2,510 (97.7)	1.00	
**Hyperlipidemia**				
yes	119 (23.2)	483 (18.8)	1.30 (1.04–1.64)	0.023
no	395 (76.8)	2,087 (81.2)	1.00	
**Obesity**				
yes	4 (0.8)	21 (0.8)	0.95 (0.33–2.79)	0.928
no	510 (99.2)	2,549 (99.2)	1.00	

SSHL: sudden sensorineural hearing loss. 95% CI: 95% confidence interval. CAD: coronary artery disease.

Table 3 shows the results of the two multivariate logistic regression analyses for SSHL adjusted for other covariates. Similar results were obtained when the associations of SSHL preexisting sensorineural hearing impairment were adjusted with either all the covariates (adjusted OR = 6.22, *p* < 0.001) or with only hypertension and hyperlipidemia that were selected using a backward elimination procedure during the model development (adjusted OR = 6.20, *p* < 0.001).

**Table 3:** Multivariate logistic regression of patients with sudden sensorineural hearing loss and controls.

Variable	Adjusted odds ratio (95% CI)	*p* value
**Sensorineural hearing impairment, Model 1**		
yes	6.22 (4.05–9.56)	< 0.001
no	1.00	
**Sensorineural hearing impairment, Model 2**		
yes	6.20 (4.05–9.48)	< 0.001
no	1.00	

95% CI: 95% confidence interval.

Model 1 adjusted for age, sex, urbanization level of patient's residence, tertile of insured amount, hypertension, diabetes mellitus, coronary artery disease or myocardial infarction, chronic kidney disease, hyperlipidemia, and obesity. Hosmer and Lemeshow test, *p* = 0.105.

Model 2 is based on a backward elimination procedure using the *p*-value of the likelihood ratio test and adjusted for hypertension and diabetes mellitus. Hosmer and Lemeshow test, *p* = 0.340.

## Discussion

This nationwide, population-based case-control study in Taiwan showed that patients with preexisting sensorineural hearing impairment had an increased risk of SSHL. The association remained significant after adjusting a number of comorbidities. This finding suggests that common sensorineural hearing impairment may share the common etiologies and/or pathophysiological changes with SSHL, which can make the inner ear more susceptible to occurrence of SSHL. Conversely, our finding does not support the notion that hypoxia preconditioning or noise preconditioning can protect patients from subsequent SSHL.

Previous research indicated that common sensorineural hearing impairment could result from aging, noise exposure, ototoxic drug exposure, obesity, hypertension, DM, dyslipidemia, CKD, or vertebrobasilar insufficiency [10, 11, 16, 17]. Although the etiology of SSHL is still unknown, SSHL has been shown to associate with infectious diseases, otologic diseases, trauma, vascular or hematologic problems, neoplastic diseases [3], cardiovascular diseases [4], CKD, and DM [8]. While the two conditions may share a number of common etiologies, the findings from this study also suggested that preexisting sensorineural hearing impairment can be considered as a novel risk factor for SSHL.

Inner ear with preexisting sensorineural hearing impairment can have a poorer functional reserve due to angiopathy, neuropathy, lower antioxidative enzyme activities and/or higher oxidative stress [18, 19]. Therefore, patients with sensorineural hearing impairment may deteriorate more rapidly or prone to new onset of SSHL than those without hearing impairment. On the contrary, the inner ear cannot be protected from SSHL by preconditioning, which causes only sub-threshold insults to the inner ear [12, 13]. The exact mechanism involved in the development of SSHL has not yet been elucidated and further studies including potential genetic risk factors are needed.

In this study, we did not included patients with conductive hearing impairments because these diseases were not supposed to be pathophysiologically associated with SSHL. In addition, we also did not included Ménière's disease and vestibular schwannoma because these conditions were associated with recurrent SSHL [20]. Therefore, our results should not be biased by the presence of these diseases. Nevertheless, several limitations of this study merit attention. First, all diagnoses were based on ICD-9-CM codes in the claim records. Misclassification of common sensorineural hearing impairment could not be completely avoided. Nevertheless, the misclassification error should be non-differential between the cases and controls, which would tend to underestimate rather than overestimate the magnitude of the odds ratios. Second, the lack of clinical data on the audiometric data and severity of hearing loss is also an inherent limitation of analyses based on the NHIRD.

In conclusion, our study showed that preexisting sensorineural hearing impairment might be a novel risk factor for SSHL. To minimize the risk of SSHL, clinicians should pay close attention to the presence of common sensorineural hearing impairment in their patients and to advise them to avoid noise and ototoxic medications.
